# Continued administration of pembrolizumab for adenocarcinoma of the lung after the onset of fulminant type 1 diabetes mellitus as an immune‐related adverse effect: A case report

**DOI:** 10.1111/1759-7714.13065

**Published:** 2019-04-09

**Authors:** Ryuya Edahiro, Mikako Ishijima, Hiroyuki Kurebe, Kohei Nishida, Takeshi Uenami, Masaki Kanazu, Yuki Akazawa, Yukihiro Yano, Masahide Mori

**Affiliations:** ^1^ Department of Thoracic Oncology National Hospital Organization Osaka Toneyama Medical Center Toyonaka‐city, Osaka Japan

**Keywords:** Immune‐checkpoint inhibitor, ketoacidosis, non‐small cell lung cancer, thyroiditis, type 1 diabetes mellitus

## Abstract

A 61‐year‐old woman with stage IVA lung adenocarcinoma exhibited high PD‐L1 expression. Pembrolizumab was administered as second‐line therapy. She developed destructive thyroiditis and her thyroid function started to decline during the administration of three to five courses. She was subsequently diagnosed with fulminant type 1 diabetes mellitus and ketoacidosis during the eighth course and insulin treatment was initiated. Pembrolizumab remained effective and was continued for 21 courses, even after the onset of diabetes mellitus. Immune‐checkpoint inhibitor treatment can be continued with hormone replacement even after the development of type 1 diabetes mellitus as an immune‐related adverse event.

## Background

Immune‐checkpoint inhibitors (ICIs) are effective for treating metastatic non‐small cell lung cancer (NSCLC) lacking sensitizing *EGFR* or *ALK* mutations.^1^ The effectiveness of pembrolizumab as first‐line or further therapy has been shown, particularly for NSCLC with high expression levels of PD‐L1.^2^, ^3^

ICI therapy induces various immune‐related adverse effects (irAE), including type 1 diabetes mellitus (T1DM), which is relatively infrequent but difficult to treat.^4^, ^5^ Several case reports on the occurrence of T1DM as an irAE in melanoma and NSCLC patients have been published. However, the clinical course of the primary disease after the onset of T1DM resulting from ICI therapy is not well known.

We report a case of an NSCLC patient who developed fulminant T1DM and ketoacidosis following treatment with pembrolizumab who achieved partial remission that was maintained by the continued administration of pembrolizumab with insulin treatment.

## Case report

**Figure 1:**
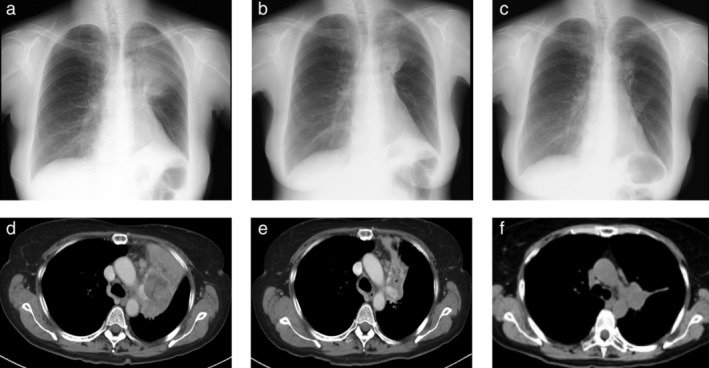
(**a**–**c**) Chest roentgenography and (**d**–**f**) computed tomography. (**a**,**d**) The primary tumor was observed in the left upper lobe before the initiation of pembrolizumab treatment. (**b**,**e**) At the onset of fulminant type 1 diabetes mellitus (T1DM) after the eighth course, the mass had reduced. (**c**,**f**) Continued pembrolizumab treatment further reduced the size of the primary lesion after the 18th course.

A 61‐year‐old female former smoker with a smoking index of 17.5 pack‐years and underlying chronic obstructive pulmonary disease, sleep apnea syndrome, and hyperlipidemia, was diagnosed with stage IVA of cT2bN1M1a (PLE) lung adenocarcinoma of the left upper lung lobe without *EGFR* mutations or *ALK* fusion (Fig 1 a,d). The PD‐L1 tumor proportion score was > 90%.

**Figure 2:**
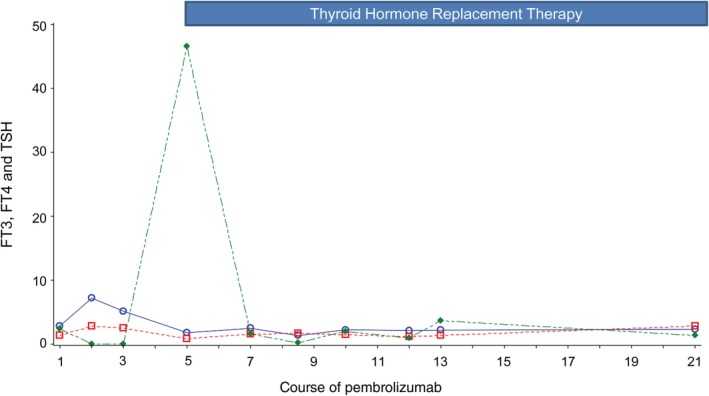
Free triiodothyronine (FT3), free thyroxine (FT4), and thyroid‐stimulating hormone (TSH) levels during pembrolizumab treatment. Thyroid hormone replacement therapy was initiated at the administration of the fifth course. 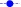 FT3 (pg/mL), 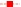 FT4 (ng/dL), 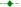 TSH (μIU/mL)

She was treated with three courses of systemic chemotherapy consisting of cisplatin and pemetrexed as a first‐line treatment, which resulted in the growth of the tumor. Three months later, pembrolizumab (200 mg/body every 3 weeks) was started as a second‐line treatment. She developed destructive thyroiditis before the third course of pembrolizumab, with her free T3 level increasing to 7.2 pg./mL and her thyroid stimulating hormone (TSH) level decreasing to 0.029 μIU/mL. At this time, she exhibited no objective symptoms; therefore pembrolizumab was continued. Before administration of the fifth course, thyroid hormone treatment was initiated because her thyroid function had begun to decline and her TSH level had increased to 46.6 μIU/mL (Fig 2).

**Figure 3:**
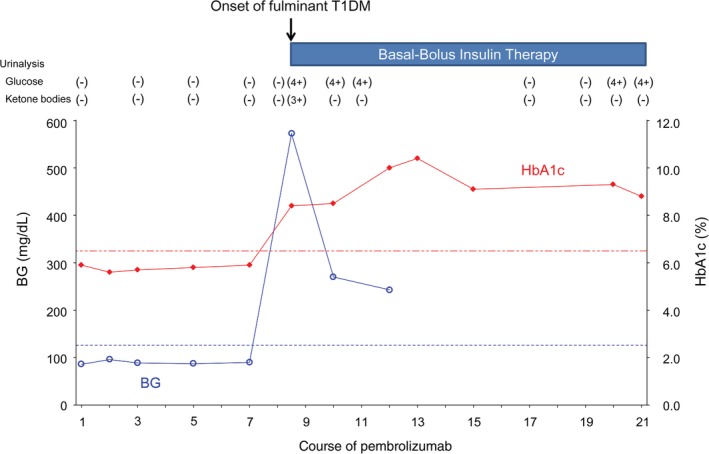
Blood glucose and hemoglobin A1c (HbA1c) levels during pembrolizumab treatment. Urinalysis of glucose and ketone bodies are also shown. The administration of the ninth course was delayed for seven days as a result of the onset of fulminant type 1 diabetes mellitus (T1DM), and the 12th course was delayed for 35 days because of acute exacerbation of chronic obstructive pulmonary disease.

Subsequently, she experienced vomiting, general malaise, and thirst from day 8 of the eighth course. She was urgently hospitalized two days later. At admission, her blood glucose level was markedly high (572 mg/dL), her hemoglobin A1c (HbA1c) level was 8.4%, her blood and urinary C‐peptide levels were remarkably low, and a urinary ketone body test was positive (Table 1). She was diagnosed with fulminant type 1 diabetes mellitus (T1DM) with ketoacidosis. After two days of fluid and electrolyte compensation and insulin therapy, her blood glucose level was well controlled and her ketoacidosis improved. Thereafter, insulin treatment for T1DM was continued (Fig 3). An anti‐glutamic acid decarboxylase (GAD) antibody test, which was performed at a later time point, was negative. She did not develop any other irAEs.

**Table 1:** Laboratory data on admission

Cell blood count				Arterial blood gas analysis (room air)	
WBC	13 800	/μL	pH	7.182	
RBC	4.07	×10^6^/μL	PaO_2_	81.7	mmHg
Hb	13.1	g/dL	PaCO_2_	19.2	mmHg
Plt	20.2	×10^4^/μL	HCO_3_^−^	6.9	mmol/L
			Base excess	−20.4	mmol/L
Biochemistry					
AST	18	U/L	Urinalysis		
ALT	14	U/L	Specific gravity	1.025	
ALP	230	U/L	Protein	(1+)	
T‐Bil	0.4	mg/dL	Glucose	(4+)	
LDH	266	U/L	Ketone bodies	(3+)	
TP	5.9	g/dL	Occult blood	(−)	
AMY	142	U/L			
Cre	0.97	mg/dL	Urine CPR	1.2	μg/day
Na	129	mEq/L			
K	3.8	mEq/L			
Cl	88.6	mEq/L			
CRP	0.91	mg/dL			
Glucose	572	mg/dL			
HbA1c	8.4	%			
CPR	0.1	ng/mL			
Ketone body fraction					
Total ketone body level	375	μmol/L			
Acetoacetate	107	μmol/L			
3‐hydroxybutric acid	268	μmol/L			
Anti‐GAD antibodies	< 5.0	U/mL			
TSH	0.19	μIU/mL			
FT_3_	1.34	pg/mL			
FT_4_	1.70	ng/mL			

AMY, amylase; ALP, alkaline phosphatase; ALT, alanine aminotransferase; AST, aspartate aminotransferase; CPR, C‐peptide immunoreactivity; Cre, creatinine; CRP, C‐reactive protein; FT3, free triiodothyronine; FT4, free thyroxine; GAD, glutamic acid decarboxylase; Hb, hemoglobin; HbA1c, hemoglobin A1c; Ht, hematocrit; LDH, lactate dehydrogenase; Plt, platelets; RBC, red blood cell; T‐Bil, total bilirubin; TP, total protein; TSH, thyroid stimulating hormone; WBC, white blood cell; γ‐GTP, γ‐glutamyl transpeptidase.

Interestingly, at the onset of T1DM, the mass in the left upper lobe decreased (Fig 1b,e). The continued administration of pembrolizumab, even after the onset of T1DM, further reduced the size of the primary lesion (Fig 1c,f). However, after 21 courses of pembrolizumab, the primary lesion alone showed slight enlargement (within the range of stable disease). The administration of pembrolizumab was interrupted and radiotherapy was performed for local control of the primary tumor.

## Discussion

Various irAEs are well known to occur in patients undergoing ICI therapy. T1DM is a relatively rare irAE, accounting for only 0.9% by nivolumab and 0.2% by pembrolizumab.^5^, ^6^ Cases in which ICI treatment leads to diabetic ketoacidosis are even less frequent. The median time to the onset of T1DM is reported to be 4.4 months; however, the time to onset ranges from 7 days to 12 months in reported cases.^5^, ^6^ Thus, it is difficult to predict the timing of the onset of T1DM. In addition, patients with T1DM induced by ICIs are often negative for pancreatic islet‐related autoantibodies (such as anti‐GAD antibody), and the disease state is not the same as general autoimmune T1DM.^7^

The diagnosis and treatment of T1DM as an irAE is the same as for general T1DM. In this case, the diagnosis itself was relatively easy, and we could manage the acute phase of fulminant T1DM and ketoacidosis with sufficient fluid supply, electrolyte correction, and the administration of insulin. Generally, many irAEs can be managed by discontinuing the ICIs or with the use of steroids; these treatments rarely improve endocrine function in cases of hypothyroidism or T1DM.^8^, ^9^ In this case, we did not use steroids as it was irreversible and the condition was manageable with insulin therapy.

Haratani *et al.* reported that the clinical effect of ICIs may be higher in patients who develop irAEs.^10^ However, T1DM did not occur as an irAE in their study, thus the association of T1DM as an irAE with ICI efficacy could not be investigated. Cases of hypothyroidism and T1DM resulting from ICI therapy can be managed with hormone replacement. As in the present case, ICI treatment can be continued after the onset of irAEs when the tumor shrinkage effect induced by ICI therapy is maintained and irAEs are manageable.

We present the case of a patient with NSCLC who developed fulminant T1DM and thyroid dysfunction as an irAE, but in whom tumor shrinkage was maintained with the continuation of pembrolizumab and the initiation of insulin therapy and thyroid hormone replacement. The further accumulation of cases is necessary to understand the clinical course and prognosis of T1DM as an irAE.
